# Malaria prevalence in Nias District, North Sumatra Province, Indonesia

**DOI:** 10.1186/1475-2875-6-116

**Published:** 2007-08-30

**Authors:** Din Syafruddin, Puji BS Asih, Isra Wahid, Rita M Dewi, Sekar Tuti, Idaman Laowo, Waozidohu Hulu, Pardamean Zendrato, Ferdinand Laihad, Anuraj H Shankar

**Affiliations:** 1 Eijkman Institute for Molecular Biology, Jalan Diponegoro 69, Jakarta 10430, Indonesia; 2 Department of Parasitology, Faculty of Medicine, Hasanuddin University, Makassar, Indonesia; 3 National Institute of Health Research and Development The Ministry of Health, Jakarta, Indonesia; 4 Nias District Health Department, North Sumatra Province, Indonesia; 5 Malaria Sub-directorate, Vector Borne Diseases Directorate, Directorate General for Communicable Diseases Control and Environmental Sanitation, the Ministry of Health, Jakarta, Indonesia; 6 World Health Organization, Jakarta, Indonesia

## Abstract

**Background:**

The Nias district of the North Sumatra Province of Indonesia has long been known to be endemic for malaria. Following the economic crisis at the end of 1998 and the subsequent tsunami and earthquake, in December 2004 and March 2005, respectively, the malaria control programme in the area deteriorated. The present study aims to provide baseline data for the establishment of a suitable malaria control programme in the area and to analyse the frequency distribution of drug resistance alleles associated with resistance to chloroquine and sulphadoxine-pyrimethamine.

**Methods:**

Malariometric and entomology surveys were performed in three subdistricts. Thin and thick blood smears were stained with Giemsa and examined under binocular light microscopy. Blood blots on filter paper were also prepared for isolation of parasite and host DNA to be used for molecular analysis of *band 3 *(SAO), *pfcrt, pfmdr1, dhfr*, and *dhps*. In addition, haemoglobin measurement was performed in the second and third surveys for the subjects less than 10 years old.

**Results:**

Results of the three surveys revealed an average slide positivity rate of 8.13%, with a relatively higher rate in certain foci. Host genetic analysis, to identify the Band 3 deletion associated with Southeast Asian Ovalocytosis (SAO), revealed an overall frequency of 1.0% among the 1,484 samples examined. One hundred six *Plasmodium falciparum *isolates from three sub-districts were successfully analysed. Alleles of the *dhfr *and *dhps *genes associated with resistance to sulphadoxine-pyrimethamine, *dhfr *C59R and S108N, and *dhps *A437G and K540E, were present at frequencies of 52.2%, 82.5%, 1.18% and 1.18%, respectively. The *pfmdr1 *alleles N86Y and N1042D, putatively associated with mefloquine resistance, were present at 31.4% and 2%, respectively. All but one sample carried the pfcrt 76T allele associated with chloroquine resistance. Entomologic surveys identified three potential anopheline vectors in the area, *Anopheles barbirostris, Anopheles kochi *and *Anopheles sundaicus*.

**Conclusion:**

The cross sectional surveys in three different sub-districts of Nias District clearly demonstrated the presence of relatively stable endemic foci of malaria in Nias District, North Sumatra Province, Indonesia. Molecular analysis of the malaria parasite isolates collected from this area strongly indicates resistance to chloroquine and a growing threat of resistance to sulphadoxine-pyrimethamine. This situation highlights the need to develop sustainable malaria control measures through regular surveillance and proper antimalarial drug deployment.

## Background

The Nias archipelago has been known to be endemic for malaria long before the natural disasters that hit the area in December 2004 and March 2005 [1,2], and to be a focus of drug resistant malaria. Both *in vivo *and *in vitro *chloroquine in Nias were described as early as 1981 [3]. Nias district was among the first locations in Indonesia where cases of chloroquine resistance in *Plasmodium vivax *were found [4,5]. Treatment failures associated with the use of sulphadoxine-pyrimethamine have also been reported in falciparum malaria cases [2]. Studies associated with the mosquito vectors revealed several anopheline mosquitoes in Nias, including *Anopheles sundaicus*, *Anopheles sinensis*, *Anopheles tessellates, Anopheles crawfordi *and *Anopheles kochi *[2,6,7], but only the first three species have been confirmed to transmit malaria. The recent monetary crisis and natural disasters have led to a deterioration of the malaria control programme in the area, culminating in the persistence of a relatively stable endemic focus and outbreak of malaria in many places after the tsunami [8].

The tsunami and the earthquake have badly affected areas along the north and west coast of the Nias archipelago. Many primary health centers and their supporting facilities were damaged. In addition, some health professionals were either killed or displaced during the two natural disasters, impairing disease control efforts in the area. In relation to malaria surveillance specifically, no primary health center in Nias District currently has any adequately trained microscopists. The present study is an effort to provide baseline data for the establishment of an appropriate malaria control programme in the area, by reviving the malaria surveillance programme in the tsunami-areas in Nanggroe Aceh Darussalam (NAD) Province and Nias District of the North Sumatra Province, using conventional and molecular tools. Results of three cross sectional malariometric surveys, host genetic analysis and entomologic surveys in Nias District, North Sumatra Province Indonesia are presented here.

## Methods

### Description of study site

**Figure 1:**
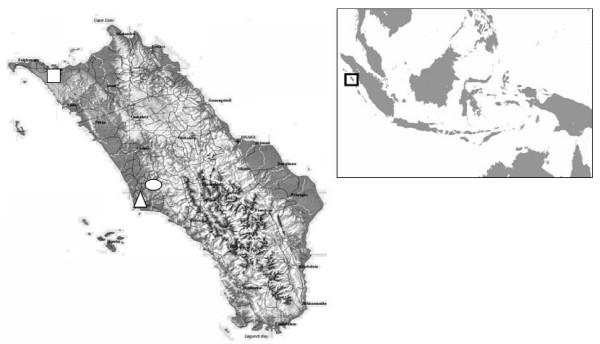
A sketch map of Nias District and its geographic location within North Sumatra Province, Republic Indonesia. The location of three selected sub-districts; Lahewa (triangle), Sirombu (square), and Mandrehe (circle) are shown.

Nias archipelago is located off the western coast of the island of Sumatra, between 0.5°–1.5° North latitude and 96° 59'–97°58' East longitude, and comprises one main island and several smaller islands (Figure 1). The administration of the islands is divided into two districts, Nias and South Nias districts. Nias district is further divided into 14 sub-districts. The Nias archipelago occupies an area of 5,625 km^2 ^with a total population of approximately 641,832 in Nias district alone. Rainfall is very high, with over three meters annually and 270 rainy days per year. The relative humidity is about 90% all year round with average temperature between 14–31°C from January to June and 22–30°C from July to December. The rainy season is from October to January, whereas the drier season starts from February to July. The main occupation of the inhabitants is farming.

### Malariometric survey

Malariometric surveillance was performed in three selected sub-districts, Lahewa, Sirombu and Mandrehe. This activity involved active and passive detection of malaria cases, using thick and thin blood smears, rapid diagnostic tests using immunochromatography test (ICT), and molecular detection using polymerase chain reaction (PCR) amplification of parasite DNA isolated from filter paper blood spots prepared from finger-pricks. For field surveys, selection of the village was based on the previous available information on malaria endemicity. All of the village inhabitants were invited to undergo parasitologic screening for malaria by submitting a drop of blood for blood smears and blot. The blood samples were considered positive if either the blood smear or the PCR was positive. Because PCR is demonstrably more sensitive than microscopy, a positive finding by PCR being consistent with a negative finding microscopy was regarded positive. During such surveys, basic 7-day morbidity history, including axial temperature and spleen grading, was recorded along with nutritional status and haemoglobin levels using a portable digital haemocytometer (Haemocue Hb201^+^, Angelholm, Sweden). In all cases, persons who were parasite positive were given appropriate drug treatment. This study received ethical clearance for the use of human subject from the Eijkman Institute Research Ethics Committee, Jakarta, Indonesia

### Entomologic survey

Collection of mosquito larvae and adults was carried out from households and adjacent areas. Adult mosquitoes were collected using light traps, capture on landing sites and human bait in- and outdoors using aspirator. Larva and/or pupae were collected in various habitats. For each habitat sampled, a collection record describing water temperature, conductivity, salinity and pH was made. Aquatic stages were transported to a laboratory and individually reared to the adult stage to determine the species using the illustrated keys of Indonesian anophelines. In addition, humidity, rainfall and average temperature were measured.

### Extraction of DNA

Parasite and human host DNA was extracted from the blood samples using chelex-100 ion exchanger (Biorad Laboratories, Hercules, CA), according to the procedure described previously [9]. The DNA was either used immediately for PCR or stored at -20°C for later analysis.

### Molecular analyses on the parasite and host

Molecular analyses were performed using PCR amplification and restriction fragment length polymorphism (RFLP) on several genes of the parasite, including *dhfr, dhps, pfmdr1, pfcrt *and of the host genes such as a *band-3 *gene deletion, indicative of Southeast Asian Ovalocytosis (SAO). The PCR reactions were carried out as previously described [10].

## Results

### Malariometric survey

The malaria prevalence in each sub-district during the three cross sectional surveys in October 2005, December 2005 and February 2006 respectively is shown in Table 1. Two species of malaria parasites, *Plasmodium falciparum *and *P. vivax *were found, mostly as single infections except in one case in Sirombu village. PCR analysis of all samples positive by microscopy and 10% randomly-chosen slide-negative samples revealed no discordance between the diagnosis by microscopy and PCR. The overall slide positivity rates were 9.9%, 6.4% and 8.1% in October 2005, December 2005 and February 2006, respectively. The vast majority of the malaria positive cases were in children less than 10 years old (Table 2). At the village level, the highest malaria prevalence was found in Marfala village in the Lahewa sub-district with a 30.5% slide positivity rate during the survey in December 2005. This prevalence decreased to 8.3% in February. In the Mandrehe sub-district, the highest prevalence was detected in the Sisarahili village with 25% slide positivity rate during the survey of October 2005.

**Table 1:** Malaria Prevalence in Lahewa, Sirombu and Mandrehe Sub-districts, Nias Districts, North Sumatra Province, Indonesia

**Sub-districts**	**Village**	**Malaria Cases**											
		
		**October 2005**				**December 2005**				**February 2006**			
		
		**Pf**	**Pv**	**Mix (Pf+Pv)**	**Number Of Slides**	**Pf**	**Pv**	**Mix (Pf+Pv)**	**Number Of Slides**	**Pf**	**Pv**	**Mix (Pf+Pv)**	**Number Of Slides**
	Lahewa	9 (5.7)	(0)	(0)	158	9 (6.6)	1 (0.7)	(0)	136	1 (1.1)	(0)	(0)	91
LAHEWA	Marfala	8 (12.7)	(0)	(0)	63	11 (30.5)	(0)	(0)	36	5 (8.3)	(0)	(0)	60
	Balafadorotuho	10 (10.3)	3 (3.1)	(0)	97	(0)	(0)	(0)	78	5 (7)	5 (7)	(0)	71
SIROMBU	Sirombu	7 (6.7)	(0)	(0)	104	9 (5.5)	1 (0.6)	(0)	162	10 (6.3)	3 (1.9)	1 (0.6)	159
MANDREHE	OnolimbuRaya	6 (7)	(0)	(0)	86	(0)	(0)	(0)	44	2 (5.5)	1 (2.8)	(0)	36
	Sisarahili	9 (25)	2 (5.5)	(0)	36	(0)	(0)	(0)	30	3 (8.1)	1 (2.7)	(0)	37
**Total individuals examined**		**49 (9)**	**5 (0.9)**	**(0)**	**544**	**29 (6)**	**2 (0.4)**	**(0)**	**486**	**26 (5.7)**	**10 (2.2)**	**1 (0.2)**	**454**

* Number in bracket indicates percentage, Pf: *Plasmodium falciparum*, Pv: *Plasmodium vivax*,

**Table 2:** Prevalence of malaria infection in each age group in Lahewa, Sirombu and Mandrehe Sub-districts, Nias District, North Sumatra Province, Indonesia

**Age group (from ≥ to <)**	**October 2005**			**December 2005**			**February 2006**		
	
	***Pf *(%)**	***Pv *(%)**	**Mix Pf+Pv (%)**	***Pf *(%)**	***Pv *(%)**	**Mix Pf+Pv (%)**	***Pf *(%)**	***Pv *(%)**	**Mix Pf+Pv (%)**
0–10	44 (8.1)	5 (0.9)	(0)	29 (6)	2 (0.4)	(0)	23 (5.1)	9 (1.9)	1 (0.2)
10–20	2 (0.4)	(0)	(0)	(0)	(0)	(0)	1 (0.2)	(0)	(0)
20–30	(0)	(0)	(0)	(0)	(0)	(0)	2 (0.4)	1 (0.2)	(0)
30–40	(0)	(0)	(0)	(0)	(0)	(0)	(0)	(0)	(0)
40–50	3 (0.5)	(0)	(0)	(0)	(0)	(0)	(0)	(0)	(0)

### Haemoglobin measurement

The haemoglobin (Hb) level was also measured in the 448 children of less than 10 years of age sampled during the second and third surveys. The relationship between Hb status and malaria infection is shown in Table 3. Among the 448 samples examined, 75 (16.7%) had moderate anaemia and three (0.67%) children had severe anaemia. Of the 75 cases that had mild and severe anaemia, six (8%) cases were found to have falciparum malaria.

**Table 3:** Haemoglobin Status and Malaria Cases in Children, In Nias District, North Sumatra Province, Indonesia

**Malaria infection**	**No. subjects**	**Hb (g %) Status**		
		
		**< 6**	**6–10**	**> 10**
Uninfected	412	2	68	342
*Pf*	26	1	5	20
*Pv*	9	-	2	7
Mixed (Pf/Pv)	1	-	-	1

Total	448	3	75	370

### Southeast Asian Ovalocytosis

The frequency distribution of SAO, a genetic disorder of red blood cells that is associated with malaria morbidity, was also checked among the samples examined. The results indicated that the overall frequency of this genetic disorder in the three selected sub-district was 1.0% (Table 4). However, at the village level, most of the cases were from the Lahewa and Sirombu villages respectively.

**Table 4:** Frequency distribution of Southeast Asian Ovalocytocis in Lahewa, Sirombu and Mandrehe Sub-districts, Nias District, North Sumatra Province, Indonesia

**Sub-districts**	**Village**	**SAO (%)**	**Total individuals examined**
	Lahewa	9 (2.3)	385
LAHEWA	Marfala	-	159
	Balefadorotuho	1 (0.4)	246
SIROMBU	Sirombu	5 (1.2)	425
	Onolimbu Raya	-	166
MANDREHE	Sisarahili	-	103

Total		15 (1)	1484

### Entomologic survey

Entomologic surveys to identify the potential mosquito vector in the surveyed area identified two anopheline species, *An. barbirostris *and *An. kochi *in Sirombu village of Sirombu sub-district. These two species were captured using light trap installed outside of houses in the village. The larval stage of both species was also found in grassy ground pools and grassy rice fields in the village. In Lahewa village, larvae of *An. sundaicus *and some other unidentified anopheline species were found in ground pools and concrete tanks, respectively. No adult anopheline mosquitoes were captured inside houses.

### Molecular assays of the *P. falciparum *isolates

In total, 109 *P. falciparum *isolates were collected during three malariometric surveys in Nias District, Indonesia. Some samples failed to amplify using certain oligos, hence the gene polymorphism information obtained was incomplete, as described below.

### Genotypic profiles of *P. falciparum *isolates from Nias

#### Polymorphisms in *pfcrt *and *pfmdr1*

Amplification of *pfcrt *among the 106 *P. falciparum *DNA isolates from Nias District was successful in 101 isolates. RFLP analysis of the amplicons revealed that all carried the 76T allele in *pfcrt*, except one isolate collected from Marfala village, which carried the wild-type allele (Tables 5, 6 and 7). In *pfmdr1*, among 102 isolates that rendered PCR products, 70 carried the wild-type allele and the remaining 32 isolates carried the 86Y polymorphism. The 1042D polymorphism was detected in two isolates, one each from Sirombu and Lahewa respectively. No polymorphisms at codons 1032 and 1246 of *pfmdr*1 were observed in any of the isolates examined.

**Table 5:** Genotypic pattern of *P. falciparum *isolates from Nias District, North Sumatra Province, October 2005

**No**	**Sample Code**	***DHFR***			***DHPS***		***Pfmdr1***		***PfCRT***
					
		***16V***	***59R***	***108N/T***	***437G***	***540E***	***86Y***	***1042D***	***76T***
1.	45L	A	-	N	G	K	N	D	T
2.	52L	-	-	-	A	K	N	N	T
3.	61L	A	R	N	A	K	N	N	T
4.	121L	A	C	S	-	-	N	N	T
5.	130L	A	-	N	-	-	N	N	T
6.	143L	A	C	S	-	-	N	N	T
7.	147L	A	-	N	A	K	N	-	T
8.	1M	A	C	S	-	-	N	N	T
9.	6M	-	-	-	A	K	N	N	T
10.	27M	A	C	N	A	K	Y	N	T
11.	37M	A	-	N	A	K	Y	N	T
12.	38M	A	C	S	A	K	Y	-	T
13.	44M	A	R	N	A	K	Y	N	T
14.	54M	-	-	-	A	K	N	-	T
15.	57M	A	C	N	A	K	Y	N	T
16.	40BT	A	R	N	A	K	N	N	T
17.	42BT	-	-	-	A	K	N	N	T
18.	44BT	-	R	-	A	K	N	N	T
19.	46BT	A	-	N	A	K	Y	N	T
20.	48BT	-	R	-	A	K	N	-	-
21.	57BT	-	-	-	-	-	-	N	T
22.	59BT	A	R	N	A	K	Y	N	T
23.	84BT	A	R	N	-	-	Y	N	T
24.	86BT	-	-	-	A	K	Y	N	T
25.	97BT	A	R	N	A	K	N	N	T
26.	2OR	-	-	-	A	K	N	N	T
27.	7OR	A	R	N	A	K	Y	N	T
28.	8OR	A	C	N	A	K	Y	N	T
29.	12OR	A	R	N	A	K	Y	N	T
30.	31OR	A	R	N	A	K	N	N	T
31.	32OR	-	-	-	A	K	N	N	T
32.	24SH	-	R	-	A	K	Y	N	T
33.	52SH	-	-	-	A	K	Y	N	T
34.	54SH	A	C	N	A	K	Y	N	T
35.	55SH	A	C	N	-	-	Y	N	T
36.	58SH	A	C	N	A	K	-	N	T
37.	59SH	A	R	N	A	K	N	N	T
38.	64SH	-	R	-	A	K	N	N	T
39.	72SH	A	C	N	A	K	N	N	T
40.	78SH	A	R	N	A	K	N	N	T
41.	2SR	-	-	-	A	K	Y	N	T
42.	3SR	A	R	N	-	-	-	N	T
43.	11SR	A	-	S	A	K	N	N	-
44.	18SR	A	C	N	A	K	N	N	T
45.	70SR	A	R	N	A	K	N	N	T
46.	81SR	A	R	N	A	K	Y	N	-
47.	103SR	A	R	-	-	-	Y	N	-

**Table 6:** Genotypic pattern of *P. falciparum *isolates from Nias District, North Sumatra Province, December 2005

**No**	**Sample Code**	***DHFR***			***DHPS***		***Pfmdr1***		***PfCRT***
					
		***16V***	***59R***	***108N/T***	***437G***	***540E***	***86Y***	***1042D***	***76T***
1.	2SR-12	A	R	N	A	K	N	D	T
2.	2SR-13	-	-	-	A	K	N	N	T
3.	2SR-28	-	-	S	A	K	Y	N	T
4.	2SR-31	-	R	-	A	K	N	N	T
5.	2SR-40	A	C	N	-	-	N	N	T
6.	2SR-46	A	C	N	A	K	N	N	T
7.	2SR-49	-	C	-	A	K	N	N	T
8.	2SR-54	-	-	-	-	-	N	N	T
9.	2SR-81	-	-	-	A	K	N	N	T
10.	2LW-5	A	-	N	A	K	N	N	T
11.	2LW-17	-	-	-	A	K	N	N	T
12.	2LW-45	-	-	-	-	-	N	N	T
13.	2LW-54	A	C	S	A	K	N	N	T
14.	2LW-77	-	-	-	-	-	N	N	T
15.	2LW-84	A	R	N	A	E	N	N	T
16.	2LW-85	-	C	-	A	K	N	N	T
17.	2LW-103	A	R	N	A	K	N	N	T
18.	2LW-107	A	C	N	A	K	Y	N	T
19.	2LW-114	A	C	N	A	K	Y	N	T
20.	2M-1	A	C	S	A	K	N	N	T
21.	2M-5	A	C	S	-	-	N	N	T
22.	2M-7	A	C	N	A	K	N	N	K
23.	2M-8	-	R	-	A	K	N	N	T
24.	2M-12	A	C	N	A	K	N	N	T
25.	2M-17	A	C	N	A	K	N	N	T
26.	2M-20	-	-	-	A	K	N	N	T
27.	2M-23	A	C	N	A	K	N	N	T
28.	2M-24	-	-	-	-	-	N	N	T
29.	2M-25	A	R	N	A	K	N	N	T
30.	2M-26	-	R	-	A	K	N	N	T
31.	2M-27	A	R	N	A	K	N	N	T
32.	2M-28	A	R	N	A	K	N	N	T
33.	2M-36	-	-	-	A	K	N	N	T

**Table 7:** Genotypic pattern of *P. falciparum *isolates from Nias District, North Sumatra Province, February 2006

**No**	**Sample Code**	***DHFR***			***DHPS***		***Pfmdr1***		***PfCRT***
					
		***16V***	***59R***	***108N/T***	***437G***	***540E***	***86Y***	***1042D***	***76T***
1.	3SR-5	A	R	N	A	K	N	N	T
2.	3SR-37	A	C	N	A	K	N	N	T
3.	3SR-47	A	C	N	A	K	N	N	T
4.	3SR-54	-	-	-	A	K	N	N	T
5.	3SR-84	A	R	N	A	K	Y	N	T
6.	3SR-86	A	R	N	A	K	N	N	T
7.	3SR-87	-	-	-	-	-	N	N	T
8.	3SR-98	-	-	-	A	K	Y	N	T
9.	3SR-121	-	R	-	A	K	N	N	T
10.	3SR-145	A	C	N	A	K	Y	N	T
11.	3SR-147	A	C	N	A	K	Y	N	T
12.	3SR-164	A	C/R	N	A	K	Y	N	T
13.	3SR-168	-	-	-	A	K	Y	N	T
14.	3SR-213	A	C	S	A	K	N	N	T
15.	3SR-223	-	-	-	A	K	Y	N	T
16.	3LW-32	A	C	S	-	-	N	N	T
17.	3LW-94	-	R	-	A	K	Y	N	T
18.	3LW-101	-	-	-	A	K	Y	N	T
19.	3LW-103	-	C	-	-	-	N	N	T
20.	3LW-107	-	-	-	A	K	Y	N	T
21.	3LW-147	-	-	-	-	-	N	N	T
22.	3LW-187	A	C/R	N	A	K	N	N	T
23.	3LW-197	-	-	-	-	-	N	N	T
24.	3LW-202	-	-	-	A	K	N	N	T
25.	3LW-203	A	C	N	A	K	N	N	T
26.	3LW-210	-	R	-	-	-	-	-	-

#### Polymorphisms in *dhfr *and *dhps*

Amplification of *dhfr *was successful with 69 isolates (Tables 5, 6 and 7). Of these, 52 isolates carried the 108N polymorphism. No 108T polymorphism was detected in any of the isolates examined. Analysis of codon 59 was successful in 69 isolates, and among these, two isolates were found to carry a mixture of both mutant and wild-type alleles. Thirty six isolates (51%) were found to carry the 59R mutant allele and the remainder the wild type 59C. No polymorphisms were observed at codons 16, 50, 51, or 164 in any of the isolates examined. Amplification of *dhps *was successful in 85 isolates and among these one isolate carried the 437G allele and one carried the 540E allele, and the remainder were wildtype. No polymorphisms were observed at codons 436, 581 or 613 in any of the isolates examined in this study.

## Discussion

The strong earthquake and the tsunami that hit the north-western coast of Sumatra and Nias in December 2004 damaged public health infrastructure in the area. This situation was worsened by the following earthquake in March 2005 in Nias, and most of the disease prevention and control measures were sidelined. Malaria was one of the major public health problems of the island long before the disasters hit the area and antimalarial drug resistance had long been documented [2,11]. Since September 2005, a bi-monthly surveillance to evaluate the post-tsunami malaria incidence in the tsunami affected areas of Nias was initiated. The results indicated a relatively stable malaria hypoendemic status in several villages of the three sub-districts Lahewa, Sirombu and Mandrehe, respectively. The findings are relatively similar to the results of previous surveys in several villages in the southern parts of the island in 1998, which are now part of the South Nias district [2]. The malaria prevalence tends to decrease in Lahewa village, which is located in the coastal area and functions as the primary town for the sub-district. This significant decrease of malaria incidence might be attributed to the intensely-conducted malaria treatment and insecticide spraying following the malaria outbreaks after the tsunami and the earthquake. However, in the other two inland villages, Marfala and Balefadorotuho, the prevalence of malaria seems to be relatively stable. There are several factors that may be associated with this situation; first, in the inland villages with a lowland forest setting, efforts to reduce the mosquito vector by insecticide spraying are difficult than on the coast, and second, malaria diagnosis and treatment may not be properly done or the parasite may have been resistant to the available antimalarial drugs. Indeed, previous surveys, have documented several falciparum and vivax malaria cases with chloroquine treatment failure [2,11].

In support of the finding, the results of the molecular analysis also indicated that virtually all of the falciparum isolates examined carried the *pfcrt *alleles associated with chloroquine resistance. With only a single exception, all of the *P. falciparum *isolates examined carried the *pfcrt *76T mutant associated with chloroquine resistance. In addition, a high proportion of the isolates also carried the 86Y allele of the *pfmdr1 *gene. The findings support the current policy to replace chloroquine with artemisinin-based combination therapy (ACT) as the first-line antimalarial treatment in the area.

Analysis of *dhfr *indicated a high proportion of isolates carrying mutant alleles associated with resistance to pyrimethamine. However, the frequency of mutant alleles of *dhfr *is slightly lower than that reported in a previous study done in the southern part of the island, but the difference is probably due to the larger sample size that was examined in this study. In the previous survey, the mutant alleles of *dhps *that associated with sulphadoxine were not found in any of the isolates examined. In this survey, two isolates that carry the 437G and 540E alleles respectively were detected. In other parts of Indonesia, mutations associated with resistance to sulphadoxine in the *dhps *gene are still relatively rare and the 437G polymorphism is the most common and earliest selected mutant allele of *dhps *[10]. In this regard, it is interesting to note that the 540E and 437G polymorphisms were not linked, as they have been suggested in previous reports [10,12].

Analysis of the frequency distribution of SAO indicated that this genetic disorder is also present in Nias population, although its frequency is much lower than that of the population in the eastern part of Indonesia where malaria is highly endemic [13,14]. Southeast Asian Ovalocytosis is a dominantly inherited haematological condition arising from the deletion of Ala^400^-Ala^408 ^in the band 3 gene [15]. The higher frequency of SAO in the population of malaria endemic area of Southeast Asia and Melanesia supports the hypothesis that this genetic defect provides relative resistance to malaria. Subsequent studies reported that erythrocytes from SAO subjects are not resistant to invasion by *P. falciparum *but that the condition is associated with protection against severe malaria in children [16,17]. The mechanism(s) by which SAO confers protection to severe malaria remains unclear but some evidence indicates that it may be associated with reduced cytoadherence [13,17]. In this regard, it is of particular interest to explore further whether the frequency of SAO, at least among the ethnic population of Indonesia, is associated with malaria endemicity.

The results of haemoglobin measurements showed a high prevalence of anaemia among children in Nias District. However, there was no significant association between anemia and parasitemia. This finding indicates that anaemia alone cannot be used as a good predictor for malaria in this area. Other possibilities including nutritional anaemia or hookworm infection should be further explored [18,19].

The results of the mosquito survey are also very similar to the previous reports that *An. sundaicus *was the predominant species along the coastal areas of Sumatra and Nias. Although in this survey only three anopheline species were detected, *An. barbirostris *that was found to breed in lowland rice field has never been reported previously [2]. The relatively few anopheline species found during the surveys is somewhat surprising as the three surveys were conducted during the rainy season. The finding may either be associated to the relatively heavier rainfall during the survey period that flushed away most of the breeding sites or it is probably biased by the limitations in sampling time and location.

In conclusion, the cross-sectional surveys in three different sub-districts of Nias District clearly demonstrate the presence of relatively stable malaria endemic foci in Nias District, North Sumatra Province Indonesia. Molecular analysis of the malaria parasite isolates collected from this area strongly indicate resistance to chloroquine and a growing threat of resistance to sulphadoxine-pyrimethamine. This situation highlights the need of developing a sustainable malaria control measures through regular surveillance and proper antimalarial drug deployment.

## Authors' contributions

DS designed the study and was responsible for data collection, management, fund raising for this study and the manuscript writing. PBSA performed the molecular analysis, data analysis, and also was involved in manuscript preparation. IW, RMD, ST, IL, WH and PZ collected field samples and performed data analysis. FL and AHS contributed to the experimental design, data analysis and the manuscript writing. All authors read and approved the manuscript.
